# Classification of functional and non-functional arm use by inertial measurement units in individuals with upper limb impairment after stroke

**DOI:** 10.3389/fphys.2022.952757

**Published:** 2022-09-28

**Authors:** Johannes Pohl, Alain Ryser, Janne Marieke Veerbeek, Geert Verheyden, Julia Elisabeth Vogt, Andreas Rüdiger Luft, Chris Awai Easthope

**Affiliations:** ^1^ Department of Neurology, University of Zurich and University Hospital Zurich, Zurich, Switzerland; ^2^ Department of Rehabilitation Sciences, KU Leuven—University of Leuven, Leuven, Belgium; ^3^ Department of Computer Science, ETH Zurich, Zurich, Switzerland; ^4^ Neurocenter, Luzerner Kantonsspital, Lucerne, Switzerland; ^5^ Cereneo, Center for Neurology and Rehabilitation, Vitznau, Switzerland; ^6^ Cereneo Foundation, Center for Interdisciplinary Research (CEFIR), Vitznau, Switzerland

**Keywords:** classification, functional, arm use metrics, inertial measurement units, thresholds, machine learning, real-life, stroke

## Abstract

**Background:** Arm use metrics derived from wrist-mounted movement sensors are widely used to quantify the upper limb performance in real-life conditions of individuals with stroke throughout motor recovery. The calculation of real-world use metrics, such as arm use duration and laterality preferences, relies on accurately identifying functional movements. Hence, classifying upper limb activity into *functional* and *non-functional* classes is paramount. Acceleration thresholds are conventionally used to distinguish these classes. However, these methods are challenged by the high inter and intra-individual variability of movement patterns. In this study, we developed and validated a machine learning classifier for this task and compared it to methods using conventional and optimal thresholds.

**Methods:** Individuals after stroke were video-recorded in their home environment performing semi-naturalistic daily tasks while wearing wrist-mounted inertial measurement units. Data were labeled frame-by-frame following the Taxonomy of Functional Upper Limb Motion definitions, excluding whole-body movements, and sequenced into 1-s epochs. Actigraph counts were computed, and an optimal threshold for functional movement was determined by receiver operating characteristic curve analyses on group and individual levels. A logistic regression classifier was trained on the same labels using time and frequency domain features. Performance measures were compared between all classification methods.

**Results:** Video data (6.5 h) of 14 individuals with mild-to-severe upper limb impairment were labeled. Optimal activity count thresholds were ≥20.1 for the affected side and ≥38.6 for the unaffected side and showed high predictive power with an area under the curve (95% CI) of 0.88 (0.87,0.89) and 0.86 (0.85, 0.87), respectively. A classification accuracy of around 80% was equivalent to the optimal threshold and machine learning methods and outperformed the conventional threshold by ∼10%. Optimal thresholds and machine learning methods showed superior specificity (75–82%) to conventional thresholds (58–66%) across unilateral and bilateral activities.

**Conclusion:** This work compares the validity of methods classifying stroke survivors’ real-life arm activities measured by wrist-worn sensors excluding whole-body movements. The determined optimal thresholds and machine learning classifiers achieved an equivalent accuracy and higher specificity than conventional thresholds. Our open-sourced classifier or optimal thresholds should be used to specify the intensity and duration of arm use.

## 1 Introduction

Motor recovery after stroke and its response to various treatment approaches have been studied thoroughly using standardized clinical assessments (Langhorne et al., 2009; Persson et al., 2016; Bernhardt et al., 2017; Dromerick et al., 2021). These assessments measure motor capacity under standardized conditions at a discrete point in time, and their relationship to real-world behavior in terms of motor performance is not fully understood (Gebruers et al., 2010). Advances in sensor technology and processing techniques have furthered the field’s understanding of how gains in motor capacity translate into real-life movement behavior throughout motor recovery post-stroke.

In the past 20 years, significant progress has been made in developing and applying clinically meaningful upper limb outcomes generated from three-dimensional acceleration signals of wrist-worn sensors during real-world upper limb use (Uswatte et al., 2000; Rand and Eng, 2012; Bailey and Lang, 2013, 2015b, 2015a; Urbin et al., 2015b; Waddell and Lang, 2018). The magnitude of acceleration (g) per time epoch(s) was unitized to activity counts (0.01664 g/s) in an early developmental stage of an activity watch manufactured for research purposes (Tryon and Williams, 1996). Activity counts, also referred to as Actigraph counts, constitute the basis for computing multiple outcome metrics that quantify the intensity, duration, and symmetry of upper limb use (Lang et al., 2017). Algorithms were made accessible to compute Actigraph counts (Brønd et al., 2017; Neishabouri et al., 2022) to harmonize the computation of sensor-based outcome metrics. This standard metric has provided an excellent basis for comparing research across studies, groups, and devices. Despite this, the refinement and validation of existing outcome metrics remain a critical goal—especially as data on validity, reliability, and responsiveness are lacking or show inconsistencies (Lang et al., 2008, 2020; Gebruers et al., 2010; Hayward et al., 2016; Waddell et al., 2017).

Content validity is defined as the degree to which a measurement instrument adequately reflects the construct to be measured (Mokkink et al., 2010). Measuring the construct of arm use is challenging. Outcome metrics are poorly specified and there is no consensus concerning the types of physical activity which should be included in their calculation. Therefore, continuous measurements typically spanning 12–24-h periods (Lang et al., 2020) contain a mix of *functional* arm movements (e.g., reaching, grasping, and transporting objects) and *non-functional* movement (e.g., walking, resting, and passive movement), whereby the latter does not per se reflect voluntary arm use. Variation of unspecified activities, including movements unrelated to functional arm use, might be inherent to fluctuations in the clinimetric properties of arm use metrics. Specification of outcome is particularly granted for metrics regarding the duration of upper limb use, where a threshold is applied to distinguish between the duration of *functional* activity, which is the outcome of interest and *non-functional* activity (inactivity). However, both *functional and non-functional *classes** contain vital information to profile physical activity in real-life conditions. More complex evaluations, aiming at extracting metrics of movement quality, typically target only the *functional* class (Okita et al., 2021; Werner et al., 2022). Quantification and quality analysis are both dependent on the accurate extraction of target classes. However, wide variation in movement patterns between individuals and throughout the rehabilitation process challenges algorithmically separating *functional* and *non-functional* movements.

The conventionally used threshold to classify functional upper limb activity was validated by video-recorded ground truth and achieved 98% accuracy in a pioneering work by Uswatte et al. classifying functional activity with >2 activity counts per 2-s epochs. (Uswatte et al., 2000; Uswatte and Hobbs Qadri, 2009). This threshold was implemented in numerous studies in slightly differing forms by using 1s-epochs and thresholds, e.g., of >2 activity counts (Bailey and Lang, 2013, 2015a; Waddell et al., 2017), ≥2 activity counts (Waddell et al., 2019), and ≥1 activity count (Urbin et al., 2015a; 2015b) for the unilateral duration of use, and >0 for bilateral and symmetry metrics (Bailey and Lang, 2013, 2015b; Urbin et al., 2015b, 2015a; Waddell et al., 2017). Consequently, a threshold of >2 per second amounts to twice that of the validated value, whereas the effect of these variations of thresholds on the validity of duration metrics remains unidentified. Furthermore, it remains unconfirmed whether the conventional threshold is valid for real-life tasks performed by individuals with different levels of motor impairment. The original study comprised a small set of tasks with defined content and duration of movement, performed by post-stroke individuals with an unknown level of impairment but the ability to perform each task without assistance (Uswatte et al., 2000). Since hemiparesis is associated with lower acceleration magnitude (de Niet et al., 2007) and spatial displacement (Vega-González and Granat, 2005) during real-life conditions, different levels of motor impairment might have resulted in distinct thresholds for the affected and unaffected upper limb. However, classifying functional upper limb movement by acceleration only might be problematic in moderate-to-severe hemiparesis since the functional activity’s lower acceleration and displacement characteristics become more similar to that of non-functional activity or inactivity.

Machine learning-based classification bears the advantage of integrating multidimensional data of additional sensing modalities (e.g., angular velocity, sensor orientation, and altimetry) registered by modern inertial measurement units (IMUs). In sensor-based movement analysis, machine learning is a powerful approach to model relations of movement characteristics in high-dimensional feature spaces (using multiple sensing modalities) to predict an outcome of interest (Jiang et al., 2017). Machine learning classifiers are being used more frequently in the field of post-stroke rehabilitation (Boukhennoufa et al., 2022), mainly targeting the recognition of physical activity types (e.g., sitting, standing, walking, and upper limb activities) (Massé et al., 2015; Boukhennoufa et al., 2021; Pohl et al., 2022) and movement classification (e.g., specific reaching patterns) (Derungs et al., 2020; Miller et al., 2020; Werner et al., 2022). Furthermore, there are a growing number of studies employing machine learning algorithms for the differentiation and prediction of functional and non-functional upper limb activities (Thrane et al., 2011; Bochniewicz et al., 2017; Lum et al., 2020). Lum et al. compared multiple machine learning classifiers and the conventional threshold method to predict semi-naturalistic functional and non-functional upper limb movement in individuals after stroke (Lum et al., 2020). While the best algorithmic classifier showed an inter-subject accuracy of 74%, the conventional threshold method (>2 activity counts) achieved poor performance as a similar acceleration magnitude was achieved in both functional and non-functional movement of the paretic limb (Lum et al., 2020). The reported accuracy of machine learning classifiers across various studies was considerably lower for the affected limb than for the unaffected and healthy controls (Bochniewicz et al., 2017; Tran et al., 2018; Lum et al., 2020). Independent of this, the relationship between clinical impairment levels and classification accuracy remains unclear.

Classification systems clearly specify movement characteristics categorizing ground truth data and, therefore, predefine the outcome to be predicted. The Functional Arm Activity Behavioral Observation System (FAABOS) was developed to validate the threshold method (Uswatte and Hobbs Qadri, 2009) and was subsequently utilized in labeling data to validate the threshold method and machine learning-based classification of functional arm use (Bochniewicz et al., 2017; Tran et al., 2018; Lum et al., 2020). According to the FAABOS, two very different movement categories, namely, motionless resting and whole body movements (such as walking), are labeled non-functional, which might significantly impact classifying by the threshold method. Since whole-body movements have recently been shown to pose bias toward metrics of unilateral symmetry of upper limb use (Regterschot et al., 2021a; 2021b), the inclusion of whole-body movements for the classification and computation of meaningful arm use outcome might be problematic. Consequently, the accurate detection of body postures—standing, sitting, and lying—within real-life conditions is a key prerequisite for computing meaningful arm use outcomes (Pohl et al., 2022). Schambra et al. (2019) proposed the “Taxonomy of Functional Upper Extremity Motion” to the facilitate machine learning-based classification of upper extremity movement split into five functional primitives differentiating purposeful movement and minimal or no motion while excluding any locomotion activities (Schambra et al., 2019). Hence, this classification system appears to reflect the measurement construct of purposeful functional arm use more adequately. In addition, it is also ideal for both threshold-based and machine learning methods to classify functional and non-functional upper limb movements. Comparing performances between both classification methods reflecting real-life activities in stroke survivors with mild-to-severe motor impairment is needed to provide ecological validity for sensor-based arm use outcomes.

Our study aims to determine the optimal thresholds of activity counts to discriminate between real-life functional and non-functional upper limb activity in individuals with mild-to-severe post-stroke motor impairment. We hypothesized that the threshold for the affected upper limb would be lower than for the unaffected upper limb and that individual thresholds would be correlated with motor impairment. Furthermore, we aimed to investigate machine learning-based classification and compare the performance against validated threshold-based methods.

## 2 Materials and methods

### 2.1 Participants

Individuals with a stroke that were followed up for a prospective longitudinal observational cohort study were invited to participate if they had mono- or hemiparesis, were able to walk independently for at least 5 m (with or without a walking aid), were living at home, and were aged above 18 years. Participants were informed regarding the goal and procedures of the study and provided written informed consent for their participation. The cantonal ethics committee in Zurich, Switzerland, provided ethical clearance to conduct the feasibility study (BASEC No. Req-2020–00947).

### 2.2 Measurement device

We used inertial measurement units (IMUs), including a 3-axis accelerometer, a 3-axis gyroscope, a 3-axis digital compass, an altimeter, a storage capacity of 4 GB, and a rechargeable battery enabling recordings of up to 72 h (https://zurichmove.com/), to record movement data. The modules were set to a sampling frequency of 50 Hz and synchronized via the corresponding docking station.

### 2.3 Procedures

A semi-naturalistic protocol was designed to record physical activities in real-world environments following Lindemann et al.’s recommendations for such procedures (Lindemann et al., 2014). Physical activities involved different body postures (lying, sitting, and standing), including uni- or bimanual activities, and walking (indoors and outdoors) and stair ascent/descent.

Participants were visited in their home environment and five movement sensors were attached, of which only wrist sensors attached to the dorsal side of the affected and unaffected wrists were relevant for this study. Physical activities were recorded using a conventional video camera (GoPro Hero7, GoPro. Inc., San Mateo, United States) with a frame rate of 30 frames per second (fps). Synchronization of start and end time between video and sensor data was obtained by videotaping the exact start time of measurements before individuals performed an instructed knocking and turning sequence of the unaffected wrist. Thereafter, participants were asked to perform tasks that are included in their typical daily activities, beginning with their morning routine, such as getting up from bed, dressing, grooming, preparing food or coffee, setting up a table, doing kitchen work, and eating. For instance, the participant was asked, “what do you typically do first after getting up?” Thus, basic daily physical activities were performed by all participants at some point, but the specific content (duration and order, and ways of how to perform activities) was open to individual preferences. In addition, participants were asked to perform their typical leisure activities which involved, for instance, desk work, handwriting, reading, watching TV, exercising, and playing a musical instrument. The individuals were asked to perform tasks as usual, whereby no instruction was given on how to use their upper extremities. We provided guidance to continue tasks in a natural manner if the storyline was interrupted or the participant was distracted by the presence of the observer.

Upper limb motor capacity was assessed by the Fugl-Meyer Assessment (FMA) upper extremity subscale (Fugl-Meyer et al., 1975) and the Action Research Arm Test (ARAT) (Lyle, 1981). Walking ability was assessed by the Functional Ambulation Categories (FAC) (Holden et al., 1984), and the ability to perform activities of daily living was assessed by the modified Rankin Scale (Dromerick et al., 2003).

### 2.4 Data processing and evaluation

**FIGURE 1:**
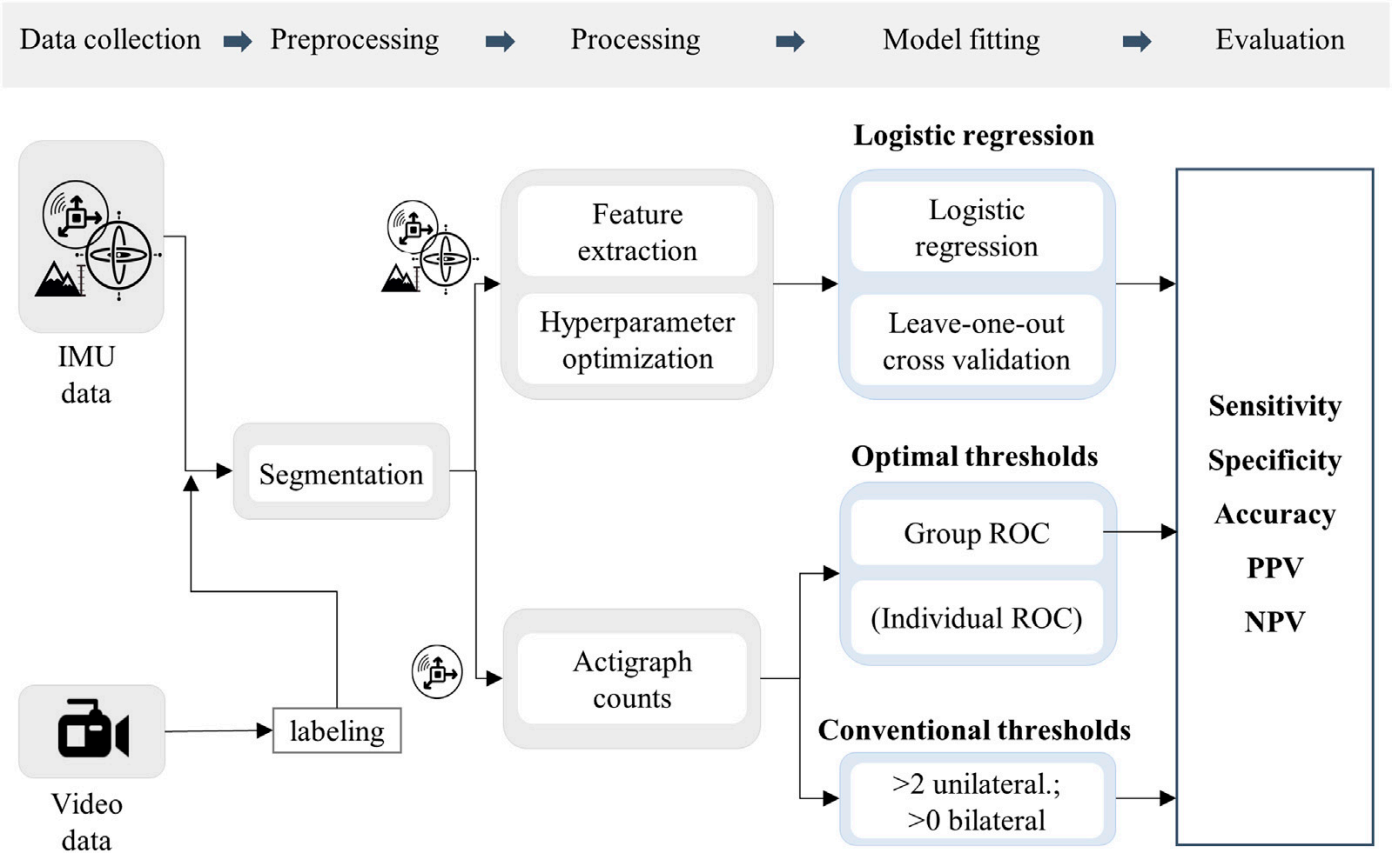
Flow chart of the methodological procedure. Icons represent the sensor modalities, accelerometer, gyroscope, and altimeter.

The preprocessing steps of data labeling and segmentation were identical for all three classification methods. The further processing steps, separated by sensor modalities and classification models, are shown in Figure 1.

#### 2.4.1 Labeling and segmentation

We labeled video data on a frame-by-frame base using Labelbox online software (Labelbox, Online, 2022. Available: https://labelbox.com) with 33.3% of data labeled by a second labeler, and agreement across labels was evaluated for quality control.

The labeling criteria for upper limb movement were in accordance with the “Taxonomy of Functional Upper Extremity Motion” (Schambra et al., 2019). The four functional primitives were collapsed into two, namely, *functional* activity and *non-functional* activity*,* which were annotated for the affected and non-affected upper limbs separately. Upper limb movement was labeled *functional* if it contained the primitives *reach* (movement with the purpose of contact with an object), *reposition* (movement toward or from an object with no contact at the endpoint), and *transport* (movement to convey an object in space). Movements that served the purpose of communication were considered *functional* (*reposition/reach*), although this task was not specifically classified in the taxonomy (Schambra et al., 2019). Minimal or no motion of the respective upper limb was labeled *non-functional,* defined by the primitives *stabilize* (holding an object still) and *idle* (steady position, no movement). Whole-body movements such as gait, transfers, and wrist movement secondary to other body parts that did not involve functional wrist movements were labeled *whole-body movements (WBM)*. We excluded these ground truth labels to reduce bias on the *non-functional* class induced by secondary wrist acceleration caused by whole-body displacement, which does not necessarily involve purposeful upper limb motor control. The labeling criteria are presented in detail in Supplemantary Table S1. Annotated labels for the body postures, lying, sitting, standing, and other activities (gait and transfers) that were used for descriptive reasons were derived from the complementing project of gait and posture detection (Pohl et al., 2022), of which algorithms can be retrieved https://github.com/StimuLOOP/activity-detection.

Finally, we labeled the sensor data by resampling the 30 fps video labels to 50 fps which corresponds to the sampling frequency of the IMU sensors.

### 2.5 Activity count thresholds

We computed activity counts with the open-source script to allow conformity of results with the widely used Actigraph counts (Brønd, 2022), which generate actigraphy counts from raw acceleration data (Brønd et al., 2017) over 1-s epochs. We computed activity counts by the vector magnitude of the three axial-accelerometer signals (axes x, y, z): $\sqrt{x^{2}+y^{2}+z^{2}}$ .

#### 2.5.1 Conventional thresholds

Following standard methods, conventional thresholds were validated against ground truth labels after dichotomization by a cut-off value > 2 activity counts for functional unilateral activity (affected side and unaffected side) and a cut-off >0 for functional bilateral activity (Bailey and Lang, 2013).

### 2.6 Optimal thresholds

Optimal thresholds for activity counts to discriminate dichotomized ground truth labels (1 = functional; 0 = non-functional) were determined for the total dataset by computing a receiver operating characteristic (ROC) separately for the affected and non-affected sides. An area under the curve (AUC) of ≥0.75 was considered clinically useful (Fan et al., 2006). The optimal threshold was set at simultaneous maximal sensitivity and specificity, thus representing the point between the most correctly classified and the least classified (Unal, 2017). Statistical software R (version 4.05) was used for statistical analysis and the *cutpointr package* (Thiele and Hirschfeld, 2021) to determine the optimal thresholds.

As an exploratory approach, we additionally computed ROC analyses on an individual level to determine the distribution of thresholds and their relationship to motor impairment as assessed by the FMA. In addition, relationships between classification accuracy and motor impairment were analyzed for each method. Interrelations were evaluated by Spearman correlations with a significance level of *p* < 0.05.

#### 2.6.1 Logistic regression

We compared movement classifications based on activity counts (optimal and conventional thresholds) with a standard model from the machine learning literature to demonstrate its advantages and applicability. More specifically, we fit a logistic regression classifier on a rich feature set extracted from the gyroscope, accelerometer, and altimeter time series of the IMU sensor. Compared to activity count thresholds, we note that this method takes more effort to apply due to hyperparameter optimization, data preprocessing, and feature extraction and it requires access to additional sensor data (gyroscope, altimeter). Labeled 1-s splits of data containing signals of the accelerometer, altimeter, and gyroscope were processed by a pipeline of filter operations and feature extraction (Figure 1) identical to those proposed in a previous work for classifying various physical activities (Moncada-Torres et al., 2014).

We processed the IMU data with consecutive filter operations to suppress noise spikes and to separate gravitational acceleration components from posture and activity data. We then extracted a set of features from the spatial and frequency domains of the signal, following the data pipeline introduced by Moncada-Torres et al., 2014. Details of the extracted features are listed in Supplemantary Table S2.

After extracting the features from all windows, we fit a logistic regression classifier to the labeled data. To optimize the model, we performed a grid search over the hyperparameter space. We evaluated each hyperparameter combination by performing leave-one-subject-out cross-validation, meaning that we partitioned validation splits by patients. We then took the hyperparameters that achieved the best accuracy during cross-validation by taking the mean over the validation splits for each laterality. To implement the machine learning method, we used Python (3.9.7) and its Scikit-learn Library (0.24.2).

#### 2.6.2 Evaluation

Classification validity of optimal thresholds, conventional thresholds, and machine learning classifier was validated with video ground truth for affected, non-affected, and bilateral activity. Bilateral activity was computed for epochs in which the affected and non-affected sides were labeled as *functional*. Bilateral activity was dichotomized (1 = functional; 0 = non-functional) using optimal thresholds (affected and non-affected), probabilistic thresholds (0.5), and conventional thresholds (>0). If both arms were considered *functional* by a given method, bilateral activity was set to *true*. If either arm was not classified as *functional*, then the label for bilateral activity was set to *false*.

Classification performance was evaluated by sensitivity, specificity accuracy, positive predictive value, and negative predictive value (Eq. 1 to Eq. 5). Due to class imbalance**,** the performance measures PPV and NPV were also analyzed by motor impairment levels. Motor impairment was categorized into three groups by established Fugl-Meyer cut-off scores: mild (43–66), moderate (29–42), and severe (0–18) (Woytowicz et al., 2017)**.**

**Sensitivity** (true positive rate, Eq. 1): the proportion of correctly classified *functional* movements amongst all *functional* ground truth labels.

$$Sensitivity=\left(\frac{true\;positives}{true\;positives+false\;negatives\;}\right).$$

**Specificity** (true negative rate, Eq. 2): the proportion of correctly classified *non-functional* movements amongst all *non-functional* ground truth labels.

$$Specificity=\left(\frac{true\;negatives}{true\;negatives+false\;positives\;}\right).$$

**Accuracy** (correct classification rate, Eq. 3): the proportion of correctly classified *functional* and *non-functional* movements amongst all *functional* and *non-functional* ground truth labels.

$$Accuracy=\left(\frac{true\;positives+true\;negatives}{true\;positives+false\;positives+true\;negatives\;+false\;negatives\;}\right).$$

**Positive predictive value** (PPV) Eq. 4: the proportion of correctly classified *functional* movements amongst all movements classified *functional.*

$$PPV=\left(\frac{true\;positives}{true\;positives+false\;positives\;}\right).$$

**Negative predictive value** (NPV); Eq5: the proportion of correctly classified *non-functional* movements amongst all movements classified *non-functional.*

$$NPV=\left(\frac{true\;negatives}{true\;negatives+false\;negatives\;}\right).$$

## 3 Results

**FIGURE 2:**
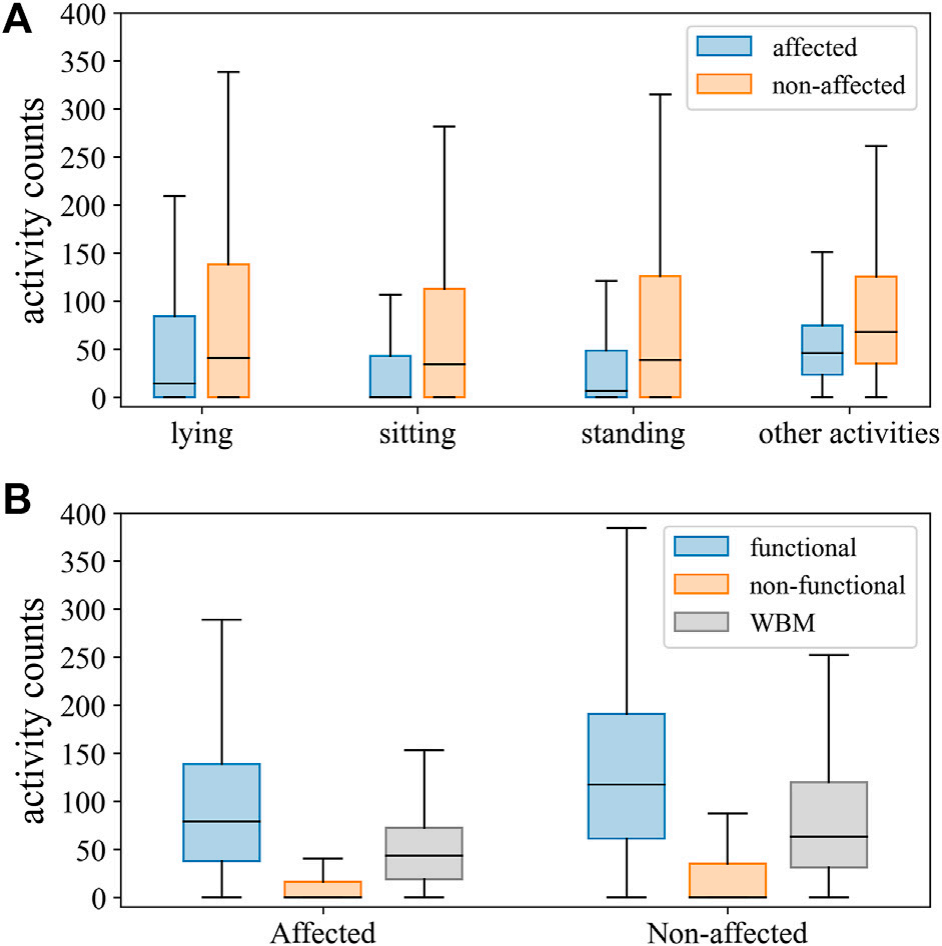
Distribution of activity counts for the affected and unaffected sides across body postures and other activities **(A)**, and upper limb movements labeled as functional, non-functional, and whole-body movements (WBM) **(B)**.

A total of 14 individuals with mild-to-severe upper limb motor impairment (Table 1) were visited in their home environment, and a total duration of 379 min of real-life activities was recorded. The agreement rate between labelers was 93.5% for all annotated labels. At the group level, the proportion of all movements labeled as *functional* vs *non-functional* was 12 versus 36%, respectively, for the affected upper limb and 23 versus 27% for the unaffected upper limb, respectively. The remaining movements were classified as *whole-body movements* and were, hence, excluded from this analysis. After removing whole-body movements, the prevalence of arm movements labeled as *functional* was low and highly variable across individuals for the affected (mean 25%; SD 19.4) and for bilateral activity (mean 20%; SD 14.9) but higher for the unaffected upper limb (mean 46%; SD 11.9). Distributions of activity counts by labels are presented in Table 2 and Figure 2. In the synthesis, we encounter an imbalance of 1:4 for *functional* versus *non-functional* labels.

**TABLE 1:** Participants’ demographic data.

Characteristics	Median	(Range)
Age	73	(50–91)
Gender (female, %)	50	—
Time post-stroke (months)	12	(4–15)
Weight (kg)	75.5	(53–95)
Height (cm)	169.5	(152–186)
Handedness (right, %)	86	—
Dominant affected (%)	36	—
FMA (/66)	38	(16–65)
ARAT (/57)	15.0	(5–57)
mRS (/6)	2	(1–3)
FAC (/5)	4	(3–5)

Legend: ARAT, Action Research Arm Test; FAC, Functional Ambulation Categories; FMA, Fugl-Meyer-Assessment upper extremity subscale; mRS, modified Rankin Scale, gender, handedness (right), and dominant affected are relative frequencies.

**TABLE 2:** Descriptives of activity counts across labels.

Label	Affected side		Non-affected side	
Body postures	n	median AC (Q1; Q3)	n	median AC (Q1; Q3)
lying^a^	1,090	14.0 (0; 84)	1,090	40 (0; 138)
sitting^a^	4,564	0 (0; 43)	4,564	34 (0; 113)
standing^a^	5,982	6.4 (0; 48)	5,982	39 (0; 126)
Other activities	10,653	44.6 (19; 85)	10,653	72 (31; 130)
Upper limb				
functional^a^	2,561	78.9 (37; 139)	5,151	118.0 (61; 191)
non-functional^a^	7,811	0 (0; 16)	5,964	0 (0; 35)
WBM	11,227	43.8 (21; 72)	10,801	65.4 (34; 122)

Legend: ^a^body postures corresponding to upper limb labels; “other activities” labels are included in whole-body movement (WBM) labels; AC, activity counts; n, frequencies of 1-s epochs, Q1, quartile 1; Q3, quartile 3.

### 3.1 Optimal thresholds

Predictive power discriminating between *functional* and *non-functional* labels was good, with an AUC (95% CI) of 0.88 (0.87, 0.89) for an optimal threshold of ≥20.1 activity counts for the affected side and an AUC (95% CI) of 0.86 (0.85, 0.87) by a threshold of ≥38.6 activity counts for the unaffected side (Figure 2). Thresholds determined on the individual level (mean threshold (SD) of 23.9, SD 13.8) were not significantly correlated to the FMA scores (*ρ* = -0.14, *p* = 0.64). The classification performance of thresholds optimized for each individual is presented in the online Supplemantary Figure S1, S2, and Supplemantary Table S2.

### 3.2 Classification accuracy

**FIGURE 3:**
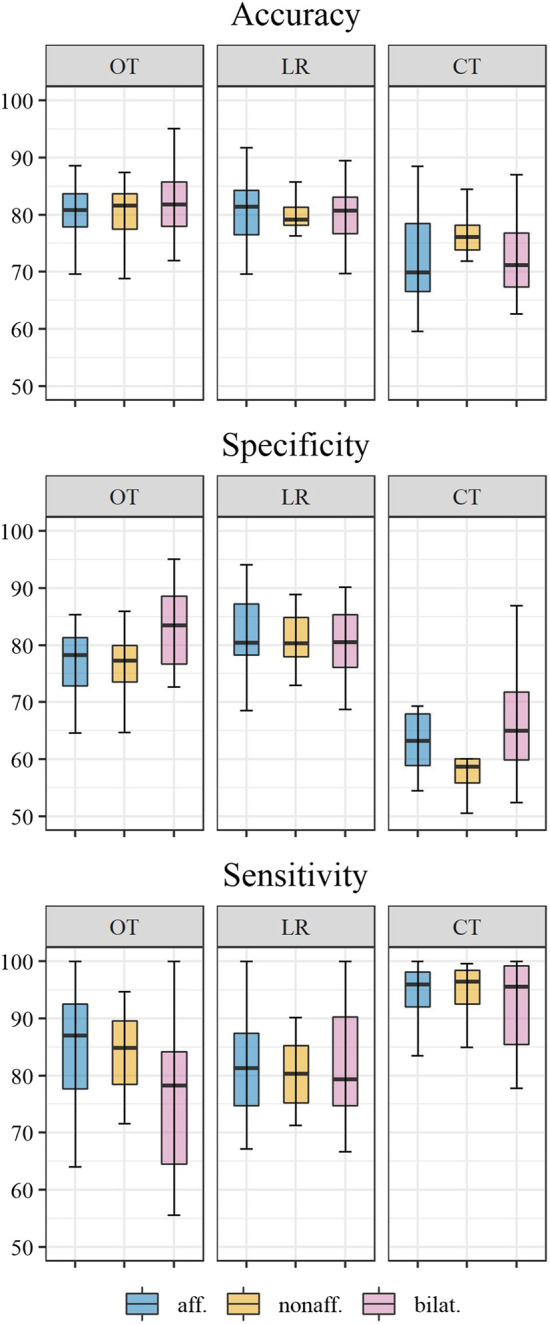
Distribution of accuracy, sensitivity, and specificity across individuals for *functional* movements of the affected side (aff., blue), non-affected side (nonaff., orange), and bilateral activity (bilat., blue). Classification methods displayed by panels: OT; optimal thresholds; LR, logistic regression; CT, conventional thresholds.

All performance measures are shown in Table 3, including unilateral and bilateral metrics for all investigated approaches. The distribution of performance is displayed by figures regarding accuracy, sensitivity, and specificity (Figure 3).

**TABLE 3:** Classification performance across methods.

Method	Laterality	Threshold	Sensitivity	Specificity	PPV	NPV	Accuracy
Optimal thresholds	Affected	≥20	85.51	77.60	55.58	94.23	79.55
	Non-affected	≥38	83.87	76.29	75.34	84.56	79.80
	Bilateral	≥20/≥38	76.08	82.33	50.66	93.52	81.13
Logistic regression	Affected	≥0.5*	78.56	79.89	56.15	91.91	79.56
	Non-affected	≥0.5*	79.46	75.05	72.26	81.71	77.04
	Bilateral	≥0.5/≥0.5*	79.13	79.41	47.81	94.10	79.35
Conventional threshold	Affected	>2	94.42	63.68	46.01	97.21	71.27
	Non-affected	>2	95.03	57.56	65.92	93.06	74.93
	Bilateral	>0	92.08	66.08	39.29	97.22	71.08

Legend: optimal thresholds on group level; ^∗^defold value of probability for logistic regression.

**FIGURE 4:**
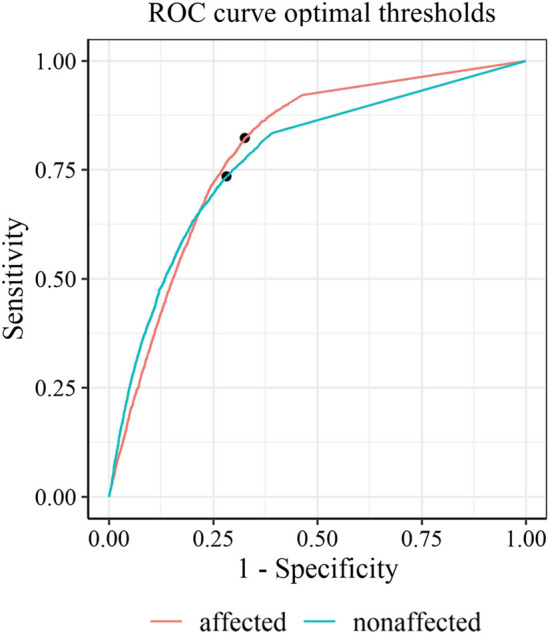
Receiver operating characteristic curves with optimal thresholds for the affected side (red) and the non-affected side (green).

Classification accuracy for unilateral and bilateral *functional* labels was similar (around 80%) for both the optimal threshold method and the logistic regression classifier (Figure 4). Minor differences between 3 and 7% were found between the optimal threshold method and logistic regression regarding their sensitivity and specificity. Accuracy was lowest for classification by the conventional threshold ≥2 activity counts across metrics (71–75%). Compared to the conventional threshold >2 activity counts, optimal thresholds resulted in lower sensitivity (by 8.9–16.0%) but higher specificity (by 13.9–18.7%) across outcome metrics.

On the individual level, classification accuracy was unrelated to motor impairment across methods (*p* > 0.05).

### 3.3 Predictive value

**FIGURE 5:**
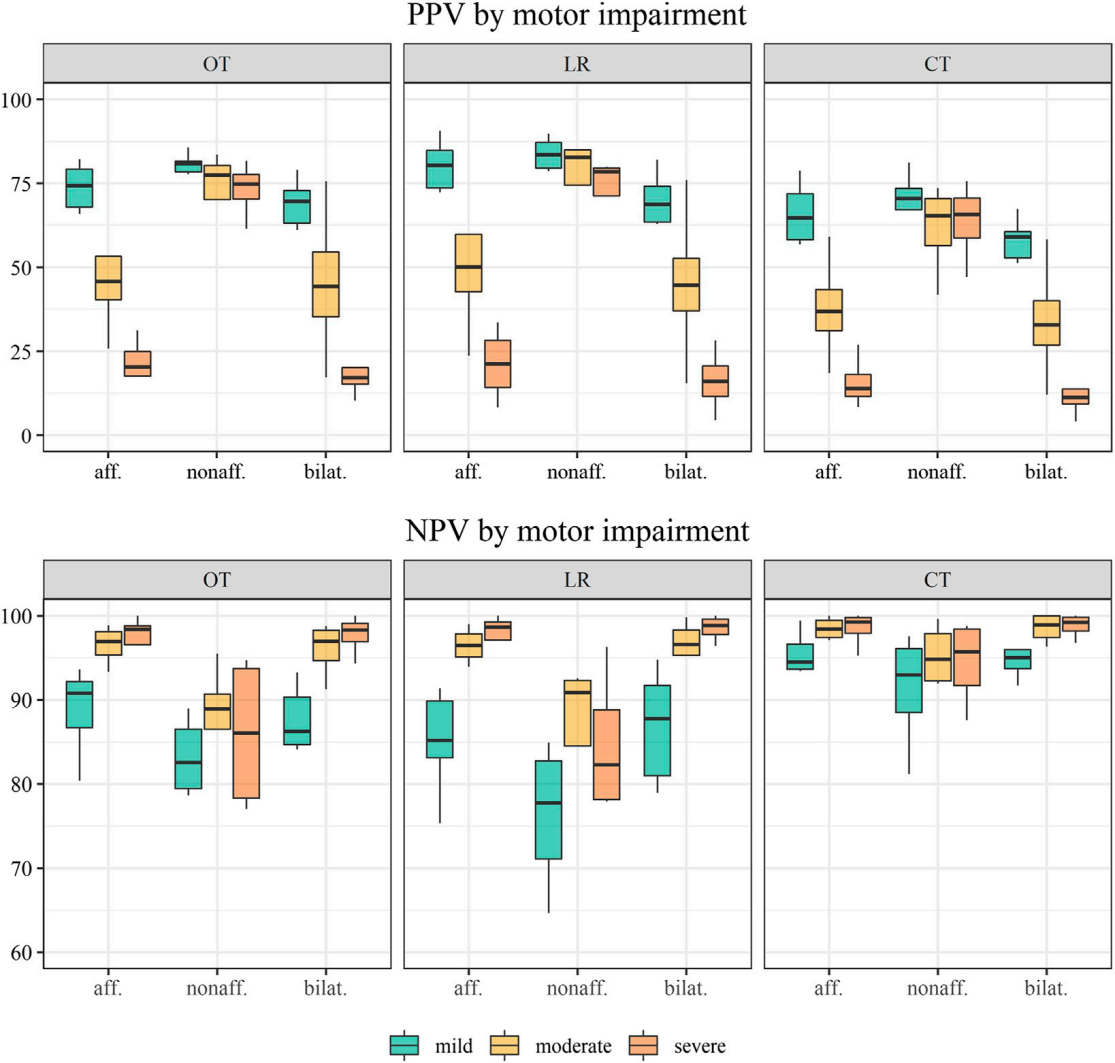
Distribution of the positive predictive value (PPV) and negative predictive value (NPV) across individuals by motor impairment levels: mild (n = 6), moderate (n = 4), and severe (n = 4). Classification methods displayed by panels: OT; optimal thresholds; LR, logistic regression; CT, conventional thresholds.

The full sample’s positive and negative predictive values are shown in Table 3 and distribution by the level of motor impairment in Figure 5. Across classification methods, total PPVs were low for *functional* movement of the affected side (46.0–55.6%) and bilateral activity (39.3–50.7%) but higher for the non-affected side (65.9–75.3). PPVs were widely distributed by levels of upper limb impairment in all classification methods. Within mildly impaired, the PPV ranged between 68.7–83.6% using optimal thresholds and logistic regression and was lower (59.0–70.5%) using conventional thresholds. The PPV decreased by the severity of impairment for functional affected and bilateral movement approximating 50% for moderate and 20% for severe impairment. The NPVs range between 81.7 and 94.1% using optimal thresholds and logistic regression but were slightly higher using conventional thresholds (93.1–97.2%). Patient variability was low, and differences by impairment were minor. Predictive values by impairment are presented in the online Supplemantary Table S3.

## 4 Discussion

Our study aimed to establish cut-off values to distinguish between real-life *functional* and non-*functional* upper limb activities in individuals with mild-to-severe motor impairment after stroke. We used labeled video ground truth data to compare movement classification from a conventional threshold-based method with two novel approaches. Specifically, one approach used only the accelerometer to compute optimal thresholds, while the second leveraged all typical sensors included in an IMU: accelerometer, gyroscope, and barometer for a machine learning approach. We examined the validity of optimal thresholds computed by both approaches for affected, non-affected, and simultaneous bilateral activity and compared classification performances to the conventionally used thresholds.

### 4.1 Activity count thresholds

We achieved good accuracy by optimal cut-offs of ≥20.1 counts per second (affected side) and ≥38.6 counts per second (non-affected side)) correctly classifying functional and non-functional activity in 80% of our data. To our knowledge, these are the first threshold values for activity counts for a wide range of movements that are validated for stroke-specific movement characteristics in real-life conditions. It is to be noted that these thresholds are one order of magnitude greater than the conventionally used threshold of 2 activity counts.

We provided full disclosure regarding the distribution of classification performance across individual subject data to facilitate the interpretation of our results. By including individuals with mild-to-severe motor impairment of the upper limb and recording habitual daily tasks in a non-scripted manner, we allowed for a natural variance of magnitude and prevalence regarding functional upper limb use. These differences in abilities by motor impairment resulted in a wide range across individuals of movements classified as *functional*, especially for the affected limb (1–63%). The described imbalance between *functional* and *non-functional* classes, exacerbated for individuals with high impairment, resulted in low PPVs independent of the applied classification method (Figure 5). Considering that data recorded in real-world settings will demonstrate similar imbalances, it is most relevant to interpret sensitivity (true positive rate) and specificity (true negative rate) (Trevethan, 2017).

The fact that the optimal threshold magnitude of the unaffected side was almost twice the magnitude of the affected side confirmed our hypothesis and aligned with previous results of overall higher acceleration magnitude recorded on the non-affected side (van der Pas et al., 2011; Rand and Eng, 2012; Strømmen et al., 2014). Accordingly, Fanchamps et al. found thresholds to differ between the affected and unaffected upper limbs using different wrist sensors and classification categories but achieved a classification accuracy of 78% for the affected and 73% for the non-affected side (Fanchamps et al., 2018). However, our results did not achieve the near-perfect classification accuracy of 98% shown by Uswatte et al. using a threshold of 2 activity counts for both the affected and non-affected upper limbs (Uswatte et al., 2000). It seems reasonable that this classification accuracy was due to precisely defined, timed, and ordered tasks delimited by periods of motionless resting. However, it remains unclear how the inclusion of whole-body movement led to a minor threshold of >2 activity counts (Uswatte et al., 2000). In contrast, we excluded labels of whole-body movement but intended to constitute real-life conditions featuring highly variable tasks and idle periods where motion was present naturally to obtain thresholds validated for the real-time setting. Considering generated acceleration magnitudes and the fact that the lowest non-zero value was 4 activity counts in our recordings, a threshold of >2 activity counts might instead classify motionless inactivity but not functionality. Therefore, the cut-off >2 activity counts showed high sensitivity, classifying every non-zero activity as functional, but low specificity classifying a large proportion false-positively as *functional*. The low specificity leads to a gross overestimation of functional activity in arm use metrics.

Pioneered by Bailey et al., the metrics’ bilateral magnitude and magnitude ratio were introduced, containing the summed magnitude of both upper limbs and the ratio between the affected and non-affected side for non-zero epochs (Bailey and Lang, 2013). We implemented optimal thresholds to investigate the validity of functional bilateral magnitude, accounting for the distinct functional contribution of the affected and non-affected sides during simultaneous bilateral activity (Bailey and Lang, 2013; Urbin et al., 2015a; Lang et al., 2017). As expected, these improved classification performances compared to the conventional threshold. Although the accuracy and specificity of functional bilateral classification were significantly higher than with a single conventional threshold, the lower and widely distributed sensitivity indicated a large proportion of false-negative classification. It is to be noted that low-level functional activities such as handwriting or typing may amount to the acceleration of magnitudes below our thresholds (Bailey and Lang, 2013) and are consequently misclassified as non-functional. However, wrist sensors are not sensitive enough to appropriately register isolated hand or finger movement; therefore, functionality cannot be classified validly (Friedman et al., 2014; Lee et al., 2019).

Functional activity can be understood as motion that occurs in the context of intention or purpose (Lang et al., 2009; Dietz and Schrafl-Altermatt, 2016; Schambra et al., 2019). Categorizing the complexity of upper limb motion into defined functional and non-functional activity classes is challenging because real-life data contain movements where intention and purpose are inconclusive. Although the clearly defined taxonomy led to high agreement between video labelers, it is inherent in the wide range of human motor behavior that a single threshold on the population level will always remain lacking without a certain level of personalization. Personalizing thresholds was possible within our annotated dataset (see Supplemantary Table S2 for details) and slightly improved classification performance. However, the magnitude of the personalized thresholds was not related to motor impairment, indicating the importance of personal movement patterns independent of the impairment level. Creating annotated ground truth data on a personalized level is extremely time-consuming and, in our opinion, not feasible for large-scale datasets. Nevertheless, the application of our proposed optimal thresholds is feasible and does not require complex classification algorithms, but it still achieved equivalent accuracy comparable to those achieved by our machine learning method.

### 4.2 Logistic regression classifier

It was our goal to compare the classification performance of accelerometer-based activity count thresholds and a machine learning-based approach using high dimensional features of additional sensor modalities (gyroscope and pressure sensor). On average, logistic regression exhibited similar classification accuracy and specificity as the activity count-based method. We observed a smaller variance in sensitivity across patients for all three metrics than in optimal thresholds, indicating robustness across stroke severity levels. The underlying lower proportion of false-negative misclassification is a major advantage compared to the acceleration-based classification.

Classifying functional upper limb movement in real-life conditions using only wrist sensors remains challenging. Thus, perfect accuracy in individuals after stroke to date remains unattained. Lum et al. (2020) conducted a validation study with a similar methodology, including a small set of semi-naturalistic tasks in individuals with stroke, achieved a lower classification accuracy of 70%, and reported large incidences of false negatively classified data (Lum et al., 2020). Other studies also reported classification accuracy between 70 and 80% (Bochniewicz et al., 2017; Tran et al., 2018) obtained by random forests, k-nearest neighbor, and support vector machine classifiers, indicating only marginal differences between classifiers for stroke individuals. However, these approaches included larger window sizes (4–5.12 s), which were associated with reduced misclassification rates (Bonomi et al., 2009). Additionally, comparison to our classification performance is limited because the mentioned studies used the FAABOS where whole-body movement and gait are classified as *non-functional* (Uswatte and Hobbs Qadri, 2009). We used the “Taxonomy of Functional Upper Extremity Motion,” which breaks down complex movements into *functional* primitives and excludes unrelated whole-body movements. Detection accuracy and extraction of whole-body movements should be considered when interpreting our results. Exemplary classification of whole-body movement including gait (e.g., over ground walking and stair ascent/descent) achieved a mean classification accuracy of 93% in real-life conditions of stroke survivors wearing additional sensors on at least one ankle (Pohl et al., 2022). Using the aforementioned taxonomy, Kaku et al. recently achieved a classification accuracy of 70% for all four *functional* movement primitives (Kaku et al., 2020). However, in their study, classification accuracy was especially low (30–40%) in patients with severe motor impairment. A low classification accuracy among individuals with moderate-to-severe impairment could be especially problematic in longitudinal investigations where the level of impairment changes by motor recovery (e.g., from severe to moderate) but the change in the classified *functional* movement would not only be due to a gain in motor ability but also due to lower misclassification probabilities. In contrast, our approach resulted in accuracies ranging from 71–95%, including moderate and severely impaired individuals.

### 4.3 Implication for thresholds in research and clinical use

The validity of sensor-based metrics builds the foundation for an increasingly large research body regarding post-stroke recovery, modeling the transfer of gains in motor capacity into real-life performance. Concurrent validity with motor capacity or perceived performance and sensor-based metrics have been shown to be low and inconsistent (Gebruers et al., 2010; van der Pas et al., 2011; Noorkõiv et al., 2014; Hayward et al., 2016).

The field of stroke rehabilitation requires valid and reliable sensor-based metrics from real-world situations to understand how interventions affect real-world behavior. In this regard, movement duration and intensity metrics are relevant, but the quality of given functional movements post-stroke is increasingly important (David et al., 2021a, 2021b; Okita et al., 2021; Werner et al., 2022). Our work strives to refine the content validity of sensor-based outcome metrics by validated classification of upper-limb movements during standing, sitting, and lying positions into *functional* and *non-functional* activity. Optimally, sequences of these body postures can be accurately detected in real-life data (Pohl et al., 2022) and extracted for subsequent classification of functional and non-functional upper limb movement. This classification can be performed for arm use outcome of intensity, duration, and symmetry domain, using thresholds or a machine learning classifier that still allows for quantifying both the *functional* and *non-functional* classes. Misclassification leads to the degradation of duration, intensity, and symmetry domain metrics. Nevertheless, applying validated functional classification for the duration, intensity, and symmetry domain metrics might positively affect the outcome’s properties.

First, duration metrics would be specified to the duration of functional use totaled within the recording length. Considering that conventional thresholds classify any registered movement as activity, the duration of functional arm use classified by our validated thresholds might lead to lower values.

Second, the mean and median of intensity metrics recorded would be shifted toward higher acceleration magnitude values by cutting off non-functional low activity. Inherent to low false-negative rates, our validated methods correctly classified 81.7–94.2% (NPV) of non-functional movements. Continuous measurements usually exhibit highly skewed distributions with disproportional high frequencies of very low acceleration magnitudes (Berkemeyer et al., 2016; Giné-Garriga et al., 2020). Hence, excluding non-functional activity might improve the responsiveness of sensor-based outcomes, which was poor in some studies (Lang et al., 2008; Waddell et al., 2017).

Third, symmetry metrics would be specified coherently since they are commonly computed by the ratio of affected (nominator) to non-affected (denominator) arm use (Bailey and Lang, 2013; Lang et al., 2017). By applying validated thresholds distinctly for the affected and non-affected bilateral arm use and symmetry, the metrics appropriately correspond to hemiparetic movement characteristics. The aforementioned arguments might be especially relevant for longitudinal investigations on the recovery of arm use and their interpretation. Recently, Lang et al. (2021) fitted a logistic regression model for duration metrics using conventional thresholds and found that recovery plateaus were reached 24 days after stroke for use duration ratio and 41 days post-stroke for paretic arm use duration (Lang et al., 2021). When considering the large proportion of false-positive classified activity by conventional thresholds shown in our results, the recovery plateau might be shifted to later time points when a validated classification is applied.

However, applying the classification of functionality comes at the cost of accepting inevitable misclassification. Functional movements of very low intensity (below thresholds) such as handwriting and typing (Bailey and Lang, 2013), or stabilizing an object with very low acceleration magnitude are misclassified as *non-functional*. Misclassification or detection failure of isolated hand and finger movements or static holding tasks should be understood as a limitation of wrist-worn sensors that are not ideal for detecting such tasks by nature. Hand and finger-mounted sensors promise the capability to detect and differentiate fine motoric hand and finger movements (Friedman et al., 2014; Kim et al., 2019; Lee et al., 2019). However, these systems remain research-grade and are not suited for mass deployment in real-world environments.

Our sample represented a wide range of motor impairments, including four severely impaired individuals with no hand function and minimal or no ability to lift their paretic arm against gravity. These individuals showed a minimal prevalence of functional movement, and the proportion of falsely positive classified incidences was high. Accordingly, amongst all movements classified as *functional,* only 20% of epochs in severely impaired and 50% of epochs in moderately impaired were indeed functional (PPV). Although the classification accuracy was independent of motor impairment, the PPVs exposed the risk of misinterpretation of sensor-based metrics in severely affected individuals. It is important to note that the magnitudes of optimal thresholds were not affected in severely impaired individuals. Removing the severely impaired individuals in a sub-analysis showed robust thresholds for the affected (≥20) and the non-affected side (≥41). However, in the absence of upper limb motor function, sensor-based arm use outcomes might contain large amounts of unrelated motion, and therefore, monitoring physical activities such as gait or sedentary time might be a preferable and more relevant outcome for this sub-group. Physical activity has been classified with high accuracy in individuals with stroke (Massé et al., 2015; Pohl et al., 2022), enabling highly specified outcome analysis and improving individualized clinical decision-making.

### 4.4 Future directions

In the course of continuous improvement of sensor-based upper limb outcomes, future work should investigate the effect of classifying functional movement on its clinometric properties. Classification by the threshold of machine learning classifiers should be applied coherently to outcome domains of intensity and duration of upper limb use. An essential refinement of outcome constitutes the extraction of periods of lying, sitting, and standing to confine upper limb outcome by excluding whole-body movement using physical activity classification algorithms. To ensure high-quality outcome measures, our classification methods should be further evaluated regarding their effect on longitudinal responsiveness and concurrent validity with benchmark clinical outcome measures.

## 5 Limitations

Our study has several limitations. First, our sample was small but contained heterogeneous motor impairments, which might have influenced the robustness of classification performance. However, this wide range of impairments was necessary to transfer our results to a representative population of individuals post-stroke. Future investigations should aim for a larger sample, including balanced numbers within motor impairment subgroups.

Second, our semi-naturalistic activity protocol contained a range of individual habitual tasks that did not include long continuous tasks of higher activity such as laundry folding or cooking. The intensity of functional activities might not have negatively influenced our threshold estimates but may have increased classification accuracy. In contrast, a more standardized protocol’s higher prevalence of functional activity could have positively influenced positive predictive values.

Consequently, future work should implement a hybrid data collection, including standardized upper limb tasks, a semi-structured protocol, and free-living conditions for the training and validation of classifiers (Allahbakhshi et al., 2018). This more complex study design has recently been shown to improve the classification accuracy in real-life conditions of elderly individuals (Allahbakhshi et al., 2020).

Third, the classification validity applies to bouts of static lying, sitting, and standing but excludes whole-body movements such as gait activity transfers and wrist movement secondary to other body parts. Therefore, end-to-end validation may require additional sensors to accurately detect and extract whole-body movements by a preprocessing pipeline. Fourth, our protocol’s magnitudes of functional and non-functional movements do not translate to continuous recordings of 24 h but represent the intensity of included tasks. Finally, we did not provide separate thresholds for bilateral functional activity. Assuming that each side could contribute differently during simultaneous activity, we determined thresholds separately for simultaneous epochs, but classification performance was equivalent. The threshold for the affected side was identical and, thus, not presented in our results. Determining thresholds for bilateral activities would optimally require a different method, including a selection of purposeful bilateral tasks, which should be addressed in future studies.

## 6 Conclusion

Wearable movement sensors can be a valuable source of information for clinical professionals regarding post-stroke individuals’ real-life movement behavior. Differentiating types of upper limb activity, such as *functional* and *non-functional* (activity versus inactivity), is paramount for quantifying arm use, and eventually, quality analysis of movements classified as functional. Popular metrics to describe arm use after stroke rely on the accurate distinction between *functional* and *non-functional* movements. Our work compares the validity of three different approaches to classifying *functional* from *non-functional* movements of the upper limb using wrist-mounted movement sensors in a stroke population in their home environment. A total of two thresholding methods were employed using activity counts for classification: traditional and optimized thresholds based on a group level for the affected and non-affected sides. We compared thresholding methods to a machine learning classifier and found equivalent classification performance between determined optimal thresholds and machine learning-based classification. Our results indicate that conventional thresholds have low specificity and tend toward overestimated activity levels. Accurate identification of functional movements is the prerequisite for quantifying upper limb use and movement quality in the next step. We here provide two validated classification methods that are accurate and easy to apply to neurorehabilitation research. These will contribute to the harmonization of calculations of upper limb outcome metrics. By providing comprehensive disclosure of classification performance and open-sourcing our dataset and algorithms, we peruse contributions to this expanding field of applied neurorehabilitation research.

## Data Availability

The datasets generated for this study and validated algorithms are available open sources: https://github.com/StimuLOOP/activity-detection.
