# Supplementary material for: Classification of functional and non-functional arm use by inertial measurement units in individuals with upper limb impairment after stroke
Source: Front Physiol. 2022 Sep 28;13:952757. doi: 10.3389/fphys.2022.952757 (PMC9554104; doi:10.3389/fphys.2022.952757)
Supplement: Supplementary file 1 [file DataSheet1.pdf]

Classification of Functional and Non-functional Arm Use by Inertial Measurement Units in Individuals with Upper Limb Impairment after Stroke

Johannes Pohl[1,2*†], Alain Ryser [3], Janne Marieke Veerbeek[4], Geert Verheyden[2] Julia Elisabeth Vogt[3], Andreas Rüdiger Luft[1,5‡], Chris Awai Easthope[6‡]

[1]Department of Neurology, University of Zurich and University Hospital Zurich, Zurich, Switzerland

[2]Department of Rehabilitation Sciences, KU Leuven—University of Leuven, Leuven, Belgium

[3]Department of Computer Science, ETH Zurich, Zurich Switzerland

[4] Neurocenter, Luzerner Kantonsspital, Lucerne, Switzerland

[5]cereneo, Center for Neurology and Rehabilitation, Vitznau, Switzerland

[6]cereneo Foundation, Center for Interdisciplinary Research (CEFIR), Vitznau, Switzerland

[†] These authors share first authorship

[‡] These authors have contributed equally to this work and share last authorship

***Correspondence:**
Corresponding Author
johannes.pohl@usz.ch

Contents

Table S1 | Labeling Classis guided by functional movement primitives 3

Table S2 | IMU features....4

Figure S1 | Receiver operating characteristic curves for the affected side....5

Figure S2 | Receiver operating characteristic curves for the non-affected side 5

Table S3 | Classification performance by individual thresholds 6

Table S4 | Predictive values by motor impairment across classification methods....7

Table S1 | Labeling Classis guided by functional movement primitives

Labels	Criteria
**Functional**	**Start:** acceleration of wrist for purposeful movement<br>**Reach:** purpose to make contact target. Motion is present. Object contact at the end may result in grasp or touch.<br>**Reposition:** purpose to move into proximity of a target although no contact to object. Can also be away from a target. Motion is present.<br>**Transport:** purpose to convey a target object in space. Motion is present. There is grasp or touch of a target object throughout the motion. Can also be scratching or sweeping.<br>***Gestures:** Movement for the purpose of communication, can be isolated, cyclic of repetitive movement.<br>**End:** start still position non-functional or WBM class
**Non-functional**	**Start:** Still position<br>**Stabilize:** purpose to hold a target object still. Motion is minimal. There is grasp of a target object throughout the minimal motion.<br>**Idle:** purpose to stand at the ready. The UE is located on a surface, at one's side, or held aloft in a stationary configuration. There is no grasp of the target object, although contact of non-target objects may be present. In these instances, idle is distinguished from the similarly appearing stabilize.<br>**End:** functional or WBM class
**Whole-body**	**Start:** wrist is accelerated primarily due movement of the whole body (trunk) in space. When feet remain in place, no purposeful movement of functional class involved.<br>Involves:<br>• Movement of wrists due to trunk movement without voluntary movement of wrist<br>• weight shift or turning while standing, arms passive<br>• Turning while lying/ sitting no arm activity involved<br>• ALL transfers and ambulation activities
**Excluded**	Arm movement cannot be labeled/ seen<br>Acceleration not due to voluntary WBM (e.g., Elevator)

Table S2 | IMU features

Domain	Feature	Acc. (post)	Acc. (act.)	Gyroscope	Altimeter
**Time**	Mean	✓			
	Standard Deviation	✓	✓	✓	✓
	Variance	✓	✓	✓	✓
	Inter-quartile range	✓	✓	✓	✓
	Percentile (3,10,20,97)	✓	✓	✓	✓
	Peak to peak amplitude	✓	✓	✓	✓
	Mean peak to peak amplitude	✓	✓	✓	
	Excessive kurtosis	✓	✓	✓	
	Slope				✓
	Root mean square	✓	✓	✓	✓
	Signal magnitude area		✓		
	XY correlation		✓		
	YZ correlation		✓		
	XZ correlation		✓		
**Frequency**	Maximal frequency component		✓		
	Energy		✓	✓	
	Entropy		✓	✓	
	Excessive kurtosis		✓		

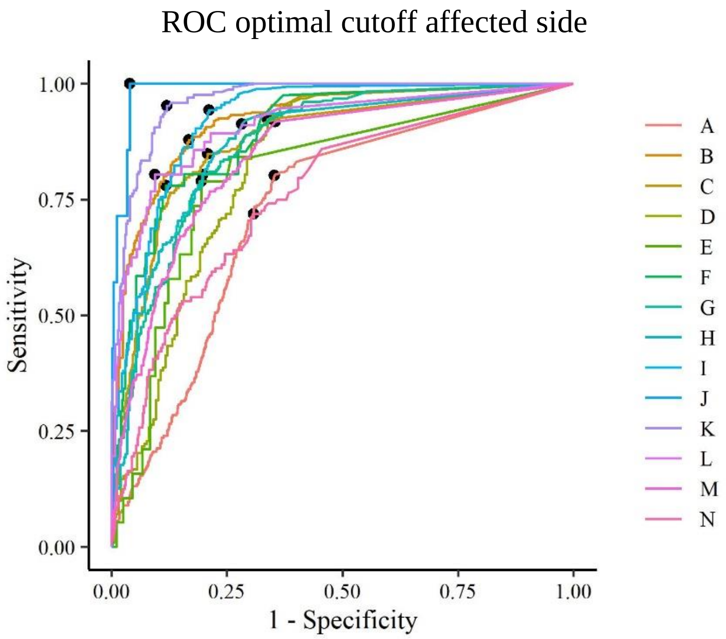

Figure S1 | Receiver operating characteristic curves for the **affected side** by individuals A to N

<

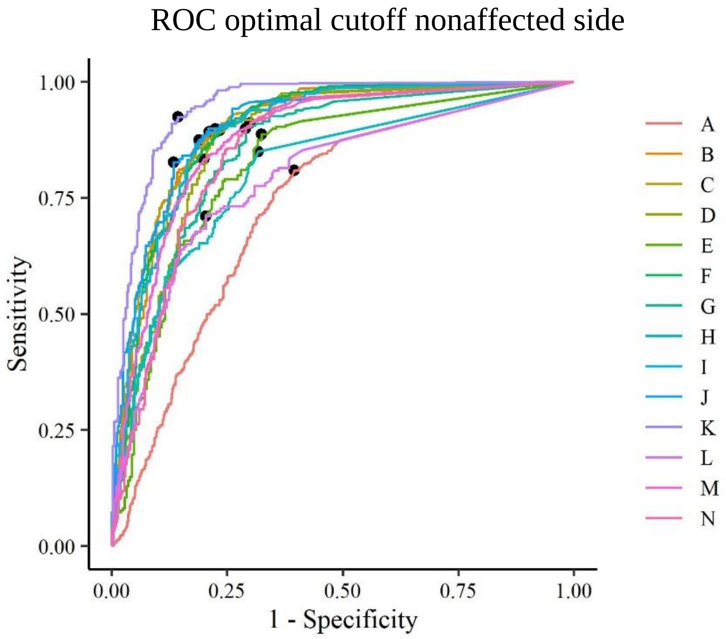

Figure S2 | Receiver operating characteristic curves for the **non-affected** side by individuals A to N

Table S3 | Classification performance by individual thresholds

#	Side	FMA	Th	AUC	Prev	Sens	Spec	PPV	NPV	Acc
**A**	aff	55	11.0	0.73	20.1	80.1	64.8	36.4	92.8	67.9
	nonaff		24.4	0.73	35.6	80.9	60.5	53.1	85.1	67.8
**B**	aff	44	38.0	0.92	54.1	87.9	83.3	86.1	85.4	85.8
	nonaff		53.2	0.90	64.2	89.2	78.9	88.3	80.4	85.5
**C**	aff	36	21.0	0.87	17.8	84.8	79.1	46.9	96.0	80.2
	nonaff		32.2	0.90	41.8	89.9	77.5	74.2	91.4	82.7
**D**	aff	57	18.4	0.83	63.3	92.1	66.2	82.4	82.9	82.6
	nonaff		41.8	0.87	48.1	89.0	77.7	78.7	88.4	83.1
**E**	aff	19	17.0	0.80	5.0	73.7	80.5	16.7	98.3	80.2
	nonaff		15.0	0.83	49.8	88.7	67.6	73.1	85.8	78.1
**F**	aff	22	35.7	0.89	6.4	78.0	88.2	31.1	98.3	87.6
	nonaff		57.6	0.90	46.8	89.3	76.6	77.1	89.0	82.5
**G**	aff	50	44.7	0.87	42.5	80.4	80.2	75.0	84.7	80.3
	nonaff		26.8	0.85	57.6	89.7	71.0	80.8	83.5	81.8
**H**	aff	65	5.7	0.87	41.2	90.9	71.8	69.4	91.9	79.7
	nonaff		4.0	0.81	51.3	84.9	68.3	73.8	81.2	76.8
**I**	aff	46	18.8	0.92	39.1	93.8	78.8	74.0	95.2	84.7
	nonaff		46.3	0.90	50.0	87.5	81.0	82.2	86.6	84.3
**J**	aff	16	31.2	0.99	1.1	85.7	96.1	18.8	99.8	96.0
	nonaff		42.3	0.90	21.8	82.7	86.5	63.2	94.7	85.7
**K**	aff	40	24.7	0.97	31.0	94.7	88.0	78.0	97.4	90.1
	nonaff		55.8	0.95	42.3	92.5	85.6	82.5	93.9	88.5
**L**	aff	35	48.4	0.91	8.0	80.4	90.6	42.5	98.2	89.8
	nonaff		40.2	0.79	25.9	71.0	79.4	54.6	88.7	77.2
**M**	aff	31	4.0	0.85	19.3	91.9	64.6	38.3	97.1	69.9
	nonaff		41.2	0.88	55.9	83.3	80.1	84.1	79.0	81.9
**N**	aff	21	16.5	0.76	16.2	71.9	69.2	31.2	92.7	69.7
	nonaff		20.6	0.86	56.1	90.7	70.0	79.5	85.5	81.6

Legend: Classification performance with individual optimal thresholds for the affected (aff) and nonaffected (nonaff) upper limb in participants (A to N); AUC, area under the curve; Acc, accuracy; FMA, Fugl-Meyer Assessment Score; NPV, negative predictive value, PPV, positive predictive value, Prev, prevalence, Sens, sensitivity, Spec, specificity, Th, threshold in activity counts,

Table S4 | Predictive values by motor impairment across classification methods

Impairment	Method	Side	PPV		NPV	
			Median	Q1; Q3	median	Q1; Q3
**Mild**	Optimal thresholds	aff.	74.3	(67.9; 79.3)	90.8	(86.7; 92.1)
		nonaff.	81.0	(78.5; 81.7)	82.5	(79.4; 86.5)
		bilat.	69.7	(63.2; 73.0)	86.3	(84.7; 90.3)
	Logistic regression	aff.	80.5	(73.7; 84.9)	84.1	(77.3; 88.7)
		nonaff.	83.6	(79.6; 87.2	77.8	(71.1; 82.7)
		bilat.	68.7	(63.6; 74.1)	87.8	(81.0; 91.7)
	Conventional thresholds	aff.	64.8	(58.2; 72.0)	94.5	(93.6; 96.6)
		nonaff.	70.5	(67.2; 73.6)	93.0	(88.5; 96.1)
		bilat.	59.0	(52.9; 60.6)	95.0	(93.7; 96.0)
**Moderate**	Optimal thresholds	aff.	45.8	(40.4; 53.4)	96.9	(95.3; 98.1)
		nonaff.	77.6	(70.2; 80.5)	88.9	(86.5; 90.7)
		bilat.	44.5	(35.4; 54.6)	97.0	(94.7; 98.3)
	Logistic regression	aff.	50.1	(42.9; 59.9)	96.5	(95.1; 97.8)
		nonaff.	82.7	(74.5; 85.1)	90.8	(84.5; 92.3)
		bilat.	44.7	(37.1; 52.7)	96.6	(95.3; 98.3)
	Conventional thresholds	aff.	36.8	(31.2; 43.5)	98.4	(97.4; 99.5)
		nonaff.	65.5	(56.6; 70.5)	94.8	(92.3; 97.9)
		bilat.	33.0	(26.8; 40.2)	98.9	(97.4; 100.0)
**Severe**	Optimal thresholds	aff.	20.3	17.7; 25.0)	98.4	(96.6; 98.8)
		nonaff.	74.9	(70.4; 77.7)	86.1	(78.3; 93.7)
		bilat.	17.2	(15.3; 20.2)	98.3	(96.9; 99.1)
	Logistic regression	aff.	21.4	(14.3; 28.2)	98.7	(97.1; 99.3)
		nonaff.	78.5	(71.3; 79.7)	82.3	(78.2; 88.8)
		bilat.	16.0	(11.6; 20.6)	98.8	(97.8; 99.6)
	Conventional thresholds	aff.	13.9	(11.6; 18.1)	99.3	(97.9; 99.8)
		nonaff.	65.8	(58.7; 70.6)	95.7	(91.7; 98.4)
		bilat.	11.2	(0.3; 13.9)	99.2	(98.2; 99.8)

Legend: aff., affected side, nonaff., nonaffected side; bilat. bilateral activity, NPV, negative predictive value; Q1, quartile 1, Q3, quartile 3; PPV, positive predictive value
